# Common Host-Derived Chemicals Increase Catches of Disease-Transmitting Mosquitoes and Can Improve Early Warning Systems for Rift Valley Fever Virus

**DOI:** 10.1371/journal.pntd.0002007

**Published:** 2013-01-10

**Authors:** David P. Tchouassi, Rosemary Sang, Catherine L. Sole, Armanda D. S. Bastos, Peter E. A. Teal, Christian Borgemeister, Baldwyn Torto

**Affiliations:** 1 International Centre of Insect Physiology and Ecology (*icipe*), Nairobi, Kenya; 2 Department of Zoology and Entomology, University of Pretoria, Pretoria, South Africa; 3 Centre for Virus Research, Kenya Medical Research Institute (KEMRI), Nairobi, Kenya; 4 USDA/ARS-Center for Medical, Agricultural and Veterinary Entomology, Gainesville, Florida, United States of America; DVBNTD/CWRU/Emory University, Kenya

## Abstract

Rift Valley fever (RVF), a mosquito-borne zoonosis, is a major public health and veterinary problem in sub-Saharan Africa. Surveillance to monitor mosquito populations during the inter-epidemic period (IEP) and viral activity in these vectors is critical to informing public health decisions for early warning and control of the disease. Using a combination of field bioassays, electrophysiological and chemical analyses we demonstrated that skin-derived aldehydes (heptanal, octanal, nonanal, decanal) common to RVF virus (RVFV) hosts including sheep, cow, donkey, goat and human serve as potent attractants for RVFV mosquito vectors. Furthermore, a blend formulated from the four aldehydes and combined with CO_2_-baited CDC trap without a light bulb doubled to tripled trap captures compared to control traps baited with CO_2_ alone. Our results reveal that (a) because of the commonality of the host chemical signature required for attraction, the host-vector interaction appears to favor the mosquito vector allowing it to find and opportunistically feed on a wide range of mammalian hosts of the disease, and (b) the sensitivity, specificity and superiority of this trapping system offers the potential for its wider use in surveillance programs for RVFV mosquito vectors especially during the IEP.

## Author Summary

Enzootic transmission of arboviral diseases such as Rift Valley Fever (RVF) continues to occur at a low intensity among mosquito vectors in Kenya, which may remain undetected by most monitoring programs unless very sensitive tools are employed to detect virus activity before an outbreak occurs. Here, we report a more sensitive and mosquito-specific surveillance trapping system for RVF virus (RVFV) mosquito vectors based on mammalian-skin derived semiochemicals. We show that RVFV mosquito vectors detect similar components (heptanal, octanal, nonanal, decanal) in the skin of RVFV mammalian hosts. In field trials, each of these compounds when combined with CO_2_ increased captures of these mosquito vectors in a dose-dependent manner. Additionally, a blend formulated from optimal attractive dose of each of these compounds combined with CO_2_ significantly increased trap captures compared to control traps baited with CO_2_ alone. The four-component blend attracted multiple mosquito vectors of the disease under field conditions suggesting that a trapping system based on this formulation offers opportunity for its use as a tool for RVFV vector surveillance.

## Introduction

Rift Valley fever (RVF) is a mosquito-borne zoonosis which is of major public health and veterinary concern in sub-Saharan Africa (SSA). In the last 20 years, epidemics of the disease have occurred at irregular intervals with hundreds of thousands of infections in humans and livestock. The 1997–1998 RVF outbreak in East Africa including Kenya, Somalia, and Tanzania represents the largest outbreak of RVF infection ever recorded in SSA that affected over 100,000 humans with over 450 deaths in Kenya alone [1]. The emergence and re-emergence of the disease especially in East Africa, poses not only a huge threat to livestock, and human health, but it also represents a looming health threat likely to spread beyond Africa due to global environmental, demographic and societal changes and trade [2]. Additionally, the economic losses due to zoonotic disease outbreaks can be staggering including trade sanctions, travel warnings or restrictions, animal disease control efforts such as animal culling (intentional slaughter), and declining public confidence in animal products [3]. For example, once RVF is known to be circulating in an animal herd, the World Organization for Animal Health (OIE) places a three-year export embargo on those animals.

Mosquito bites are the most important transmission mechanisms of the disease to mammalian hosts including humans [4], [5]. Although several mosquito species in diverse genera have been implicated as vectors following isolation of the RVF virus (RVFV) [5]–[8], there is strong evidence that in Kenya, *Aedes mcintoshi* and *Ae. ochraceus* play key roles in the transmission of the disease [8]–[10]. During the 2007/2008 RVF outbreak in Kenya, these two species were identified as primary RVFV vectors, accounting for over 77% of positive pools of mosquitoes sampled in the field [8] which occur predominantly in North-Eastern Kenya. In addition, *Mansonia* and other *Culex* mosquitoes are important RVFV vectors in Marigat District of Rift-Valley area, a highly endemic area of the disease [8].

In spite of the apparent emergence and re-emergence of arboviral diseases and especially RVF in East Africa, sensitive surveillance programs to actively monitor vector populations to provide an early warning system are lacking. Entomologic arbovirus surveillance is advantageous because it (i) provides the earliest evidence of transmission in an area, (ii) identifies the potential risk to humans, and (iii) allows emergency control operations to be set in motion in advance of epidemics. Vectors once infected, remain infected with the virus for the duration of their life, unlike in humans and other vertebrates which are only transiently infected [11]. The option of serologic surveillance in animals is complicated by problems of cross reactivity among arbovirus groups [12]–[14]. Moreover, other challenges that may compromise the efficacy of animal hosts as a surveillance tool include ethical issues associated with using animals; challenges of bleeding larger animals which represents an occupational health and safety issue [12]; and reduced sensitivity due to the development of herd immunity [13], [14] which may dampen seroconversion. This makes vector surveillance the best option to target for arbovirus activity especially as RVF epidemics in these susceptible animals, initiated by bites of infected mosquitoes, are also involved in sustaining the disease. Until now, RVFV vectors have been monitored using CO_2_-baited CDC light traps, which are generally non-specific and trap a wide range of non-target insect species such as beetles and moths, in addition to mosquitoes. Additionally, because of low sensitivity, this trapping system is inadequate for use during the low intensity inter-epidemic period (IEP) of enzootic virus transmission where viral activity may remain undetected among mosquito species [4], [12], [15]. Thus, there is a critical need to develop more sensitive and effective monitoring tools to increase trap captures of mosquito vectors so as to maximize detection of virus activity.

Like most hematophagous insects, RVFV vectors use olfactory cues to locate their hosts for a blood meal [16], [17] which may involve more than mammalian breath odors such as CO_2,_ a non-specific semiochemical, commonly used in the CDC light trap. We therefore refined the sensitivity of the existing trapping system for RVFV vectors by combining it with known mammalian host skin-derived semiochemicals in order to target only mosquitoes. Here we report the identification of key kairomones responsible for attraction of RVFV vectors which demonstrate the commonality of mammalian host skin-derived attractants for mosquito vectors of the disease, and development of a highly efficient monitoring tool for RVFV vectors which exploits a semiochemical lure, developed from skin odors of these mammals that can potentially impact RVFV mosquito surveillance during the IEP.

## Materials and Methods

### Study sites

**Figure 1:**
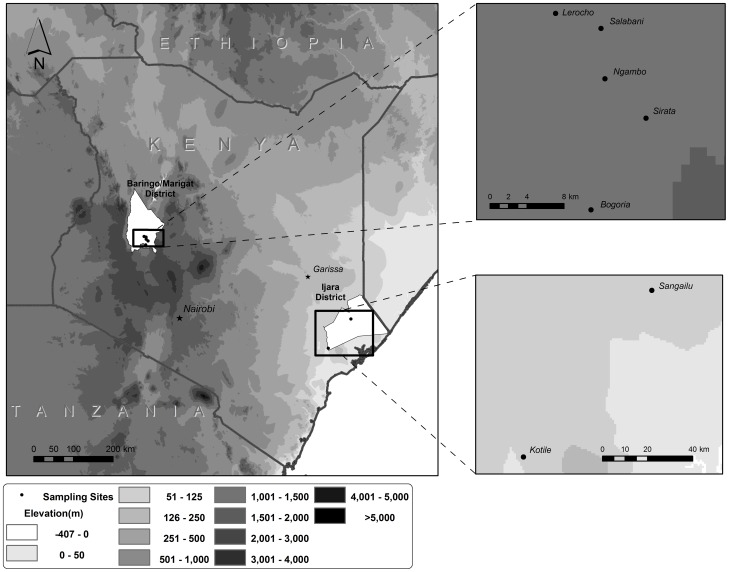
Map of Kenya showing the location of the study sites.

All experiments were carried out at two ecologically distinct sites, i.e. Ijara and Marigat districts of Kenya (Figure 1), both highly endemic areas for epidemic RVF [8] and which are currently under active surveillance for arbovirus activities.

Ijara District is located in the North Eastern Province of Kenya, where traps were set out in two major locations: Sangailu (01.31°S, 40.71°E) and Kotile (1.97°S, 40.19°E). The entire district is semi-arid and normally experiences two rainy seasons a year which frequently fail: the so-called short rains between October and December and the long rains in March and April. The area is located at an altitude of about 60 m above sea level (asl) and typical annual rainfall averages between 300 to 500 mm. The people in North Eastern Province are predominantly ethnic Somali and practice pastoralism, keeping livestock including cattle, goats, sheep, camels, and donkeys. Vegetation predominantly consists of shrubs and acacia bushes.

In Marigat District located in the Rift Valley Province of Kenya, traps were set in surrounding villages/communities namely N'gambo (0.50°N, 36.06°E), Salabani (0.55°N, 36.06°E), Lerocho (0.56°N, 36.01°E), Bogoria (0.37°N, 36.05°E) and Sirata (0.46°N, 36.10°E). The vegetation in the low lying arid part of Marigat district consists of northern Acacia-Commiphora bushlands and thickets and has experienced severe land degradation caused by uncontrolled grazing. The local inhabitants who are mainly agro-pastoralists subsist on limited crop production and livestock rearing. This district located around 1000 m asl receives annual rainfall ranging from 300 to 700 mm.

### Odor collection and field evaluation of crude skin odors of RVFV hosts on mosquito captures

**Figure 2:**
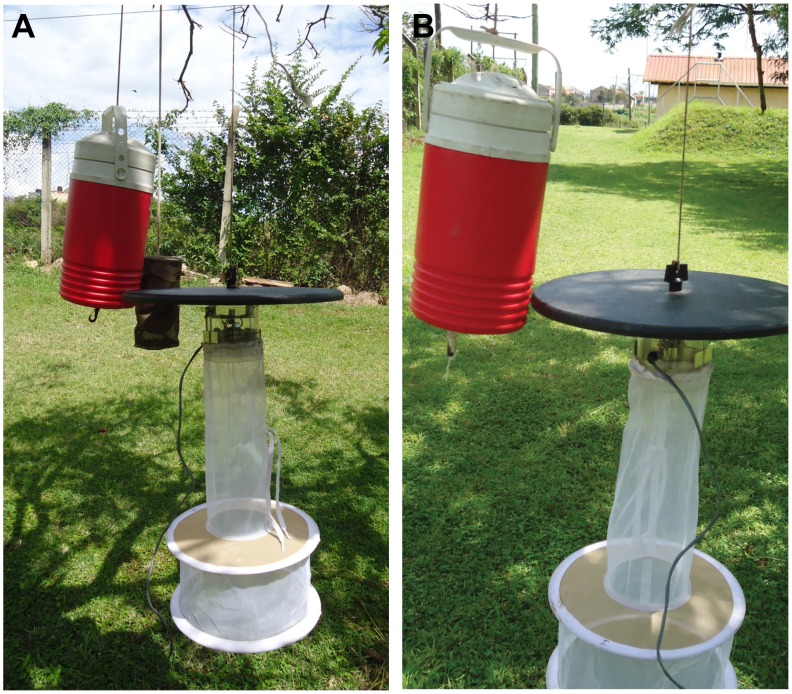
Trap design using CDC trap without a light bulb for field evaluation. (A) crude animal skin odors: arrangement of canister and CO_2_ released at the bottom of the Igloo container all placed close to the fan of the trap; (B) synthetic compounds released from a 0.5 ml tube placed under the Igloo close to the air flow of CO_2_.

Skin odors from cow, donkey, goat and sheep were collected by rubbing stockinette cotton material (Clinitex, FL Orthopedics, USA, latex free with antimicrobial protection) on the belly and back areas avoiding the head and anal regions for 12–15 minutes using 4 pieces of the material per animal each measuring 10 cm×26.5 cm. The stockinette material was handled with latex gloved hands in order to minimize contamination from human skin. Human odor used in the experiment consisted of four pieces of worn stockinette (of same material and sizes as used for the animals) containing trapped foot odors from a 30-year old African male volunteer by wearing them for 20 hours (21:00–17:00 the following day) prior to the start of the experiments. Stockinette materials with animal odors were wrapped in at least four layers of aluminium foil, kept in a cold box (10°C) and immediately transported to the trapping site. Once at the trapping site, the stockinette human and animal odors were placed in separate canisters (cylindrical in shape with diameter 9.5 cm and height 22.5 cm) designed from Brass mesh wire (mesh size, 0.006 Inch, McNichols, Tampa, FL) and hung close to the air flow of CO_2_ released at the bottom of an Igloo thermos container both mounted close to the fan of the CDC trap without a light bulb (Figure 2). All canisters made from the same material and of similar size, were boiled in l0% bleach solution after each night's trapping to eliminate any residual odor. The stockinette material containing collected odors were replaced each day for a repeat of the experiment. In total, six treatments were tested consisting of animal skin odor of each animal type+CO_2_ (five treatments) and CO_2_ only. Carbon dioxide was added nightly and delivered by placing 1 kg dry ice in Igloo thermos containers (2 L) (John W Hock, Gainesville, FL) with a 13-mm hole in the bottom center. With an inter-trap distance of 40 m, the treatments and control were randomly assigned to a predetermined similar area following a Latin square design with days as replicates. Traps were activated within 30 min of sunset (1800–1830 hr) and trap contents collected within 30 min after sunrise (0600–0630 hr). Traps were rotated on every trapping day to minimize variability due to trap placement. The field experiments were conducted at Ijara and Marigat districts which are two highly endemic areas for RVFV activities in Kenya. The ‘attraction’ of animal/human odor was estimated by the number of mosquitoes collected from the CO_2_-baited CDC trap (model 512, John W Hock, Gainesville, FL) without a light bulb containing the bait odor from that animal compared to the control (CO_2_ alone without a light bulb only) in several replicate exposures. Mosquitoes were morphologically identified to species using taxonomic keys [18]–[20]. Mosquitoes were categorized as engorged when blood fed or gravid based on observation of their abdominal condition as illustrated in the WHO Manual [21]. Daily counts of number of mosquitoes per treatment were analyzed using a generalized linear model (GLM) with negative binomial error structure and log link in R 2.11.0 software [22]. Using the CO_2_ baited CDC trap (control) as the reference category, the incidence rate ratios (IRR), a likelihood measure, that mosquito species chose other treatments instead of the control were estimated including Confidence Interval (CI) and corresponding P-values. The IRR for the control is 1 (unity) and values above this indicates better performance and values below under performance of the treatments relative to the control. Chi-square goodness-of-fit was used to analyze the effects of odors on the proportion of total engorged mosquito (i.e., total counts of blood fed+gravid mosquitoes in the total captures) recorded for each trap treatment. Also, a pair-wise test of significant differences in the proportions of engorged mosquitoes between each of the combined CO_2_+animal odor treatments relative to the control (CO_2_ only) was performed using chi-square goodness-of-fit. Another measure was derived based on the ratio of these proportions for each of the animal odors relative to the control. This measure is termed the catch index; odors which, say, double or halve the catch from a trap would have catch indices of 2 and 0.5, respectively.

### Collection of stockinette trapped odors

We collected headspace odors (24 hrs) from stockinette samples brought from the field under cold storage on Super Q adsorbent (30 mg, Alltech, Nicholasville, KY) and eluted filters with 200 µl dichloromethane (DCM)/hexane mixture (50∶50). Headspace trapping was performed using the Volatile entrainment system using the trapped odors on 4 pieces of stockinette material of equal size taken per animal. All the eluents were concentrated under nitrogen to 100 µl and followed by GC-EAD and GC-MS analyses.

### Coupled gas chromatography electroantennographic detection (GC-EAD) and coupled gas chromatography mass spectrometry (GC-MS) analyses of odors

Mosquito head excised with a scalpel from adult female mosquitoes (aged 5–8 days, for laboratory-reared mosquitoes) were used for electrophysiological recordings. Similar preparations were made for wild caught adult mosquitoes (age unknown for field collected insects) trapped with CO_2_-baited CDC miniature light trap and maintained on 6% glucose solution. The excised head was mounted between two glass capillary (1.1 mm I.D.) electrodes filled with Ringer solution (prepared by dissolving 6.5 g NaCl, 0.42 g KCl, 0.25 g CaCl_2_ and 0.1 g NaHCO_3_ in one liter of distilled water). Silver–silver chloride junctions were used to maintain electrical contact between the electrodes and input of preamplifier (10A; Syntech, Hilversum, The Netherlands). The grounded reference (indifferent electrode) was connected to the base of the antenna, and the tip connected to the recording electrode. The analog signal was detected through a probe (INR-II, Syntech, Hilversum, The Netherlands), captured and processed with a data acquisition controller (IDAC-4, Syntech, The Netherlands) and into a personal computer. Recordings were later analyzed with software (EAG 2000, Syntech, Hilversum, The Netherlands).

In GC-EAD analysis, 2 µl of the odor extract was injected into a GC linked to the antenna recording setup. Injections of the extracts were conducted on a HP 5890 gas chromatograph fitted with a splitless injector (220°C) and flame ionization detector (FID) (280°C). Compounds were separated on a nonpolar capillary column HP-1 (30 m×0.25 mm×0.25 µm film thickness) with nitrogen as the carrier gas. The oven temperature was held at 35°C for 5 min and then increased at 10°C/min to a final temperature of 280°C, which was held for 10 min. The GC was fitted with a split at the end of the column, delivering half the column effluent to the flame ionization detector (FID) and the other half to a humidified airstream (1 ml/min) flushing over the antenna via a heated transfer line (260°C) (Syntech). Commercial authentic standards were used to confirm EAG activity of tentatively identified components following similar procedures.

For GC-MS analysis, 1 µl of volatile extract from the different animals were analyzed on an Agilent system consisting of a model HP 6890A gas chromatograph, a model 5973 mass selective detector (EIMS, electron energy, 70 eV), and an Agilent ChemStation data system. The GC column was an HP-5 ms fused silica capillary with a (5% phenyl)-methylpolysiloxane stationary phase (30 m×0.25 mm×0.25 µm film thickness). The carrier gas was helium with a column head pressure of 7.07 psi and flow rate of 1.0 ml/min. Inlet temperature was 200°C and MSD detector temperature was 280°C. The oven temperature was held at 35°C for 2 min and then increased at 10°C min^−1^ to a final temperature of 280°C, which was held for 10 min. The identity of EAG-active compounds was determined by comparison with references from mass spectral libraries (NIST05, Agilent Technologies [NIST database, G1036A, revision D.01.00, ChemStation data system (G1701CA, version C.00.01.08). Final confirmation of identity was achieved by coinjection with synthetic reference compounds. Additionally, trapped odors from unused cotton material were included as control in all GC/MS analysis. Solvent blanks (hexane or DCM) were concentrated and analyzed to identify contaminants. The blanks were analyzed as described for the samples. Compounds present in the blank analyses were excluded from the composition percentages of compounds in the samples. To estimate ratio of abundance of aldehyde components, in each GC-MS run, the percent composition of each of the aldehyde components in the overall chemical profile of headspace odor from each animal was recorded. The percent abundances based on peak areas from an integrated chromatogram were then used to establish mean ratio of one component in relation to the other for each animal after 3 replicate runs (Table S1). The ratios of synthetic blends were also compared to the ratios in the naturally occurring blends. Similar chromatographic data were used to estimate release rates of the constituent aldehydes from each of the animals (Table S2) by recording the peak area of each constituent obtained from an integrated chromatogram. External quantification was done using authentic sample of nonanal. Peak areas were recorded for different known concentrations of nonanal covering the expected analyte concentration range and a calibration curve and subsequent linear equation obtained which was then used to estimate the amount or quantity of each component produced following GC-MS analysis of crude animal volatiles. Estimated release rate was calculated taken into consideration the trapping duration, total volume of sample eluted in solvent (dichloromethane) and quantity analyzed by GC-MS (1 µl).

### Field evaluation of identified components on mosquito captures

Heptanal, octanal, nonanal, decanal (>98% pure, Sigma-Aldrich) were formulated in hexane at different concentrations with antioxidant, 2,6-di-*tert*-butyl-4-methylphenol (butylated hydroxytoluene, BHT, Aldrich) added for field evaluation. Initial field assessments of lures at *icipe*'s Duduville Campus, followed by GC-MS analysis showed that the aldehydes oxidized to their corresponding fatty acids after at least 18 hr exposure. Therefore for long term assay, we formulated our lures using this anti-oxidant. Two milligrams (10% of individual component) of antioxidant was added to 20 mg of each component in 1 ml of hexane to obtain a stock concentration and this was serially diluted to obtain various concentrations. Blends were constituted by mixing equal amounts of the respective components. All lures either singly or blends were released by diffusion from 0.5 ml polyethylene tubes with a pin hole in the center of the cap. Preliminary trials to determine possible range of attractive doses of compounds were conducted at *icipe*'*s* Nairobi campus (Table S3). No mosquitoes were trapped in the control CDC trap with the light bulb removed. Informed by similar response profile in all the RVFV mosquitoes, it was clear that likely attractive doses would be at most 5 mg/ml for the individual compounds. Consequently, concentrations of individual compounds, including 0.1, 0.5, 1, 2 and 5 mg/ml were evaluated in preliminary field assessments to determine optimal attractive doses of each compound in three replicate trials (Figure S1). We also conducted an analysis of the amount of aldehyde released from the different animals per hour (Tables S2 and S3). Representative blends simulating ratio of occurrence of the compounds in each of the animal odors were also evaluated. Details of these blends are shown in Table S4. Nominal release rates were measured in the laboratory at 20°C and 0.5 m/sec airflow (Table S5) by loading 0.5 ml of each compound tested in replicated dispensers and then measuring weight loss every 12 hours. Final weight loss measurements were recalculated as micrograms of compound lost per hour.

Lures were attached underneath in the airflow of CO_2_ (dry ice) released from an Igloo cooler. These were mounted close to the fan of the CDC trap without a light bulb suspended 1.5 m off the ground on a tree. Effect of individual components and blends were evaluated for mosquito trap captures in field experiments conducted in January 2012 at Ijara district following a randomized experiment in the same predetermined similar area with days as replicates. Traps were rotated on every trapping day to minimize variability due to trap placement. Mosquito captures in CDC traps without a light bulb baited with a combination of CO_2_ and different doses of individual components/blends and were compared with captures to control trap with CO_2_ alone. Traps were activated within 30 min of sunset (1800–1830 hr) and trap contents collected within 30 min after sunrise (0600–0630 hr). Daily counts of number of mosquitoes in the different trap treatments were recorded and analyzed using negative binomial regression following GLM procedures in R as described previously. Also proportion of engorged mosquitoes and catch indices for each treatment were analyzed as described previously. Data were analyzed for total mosquito captures of primary RVFV vectors (*Ae. mcintoshi* and *Ae. ochraceus*) and for total mosquito collections including other important RVFV *Culex* vectors such as *Cx. pipiens quinquefasciatus*, *Cx. univittatus*, *Cx. poicilipes*.

### Ethics statement

The study was conducted with the approval of the national ethics review committee based at the Kenya Medical Research Institute (KEMRI) and is renewed on an annual basis after a scientific audit. The Animal use component was also given approval by the KEMRI Animal Use and Care committee (KEMRI-AUCC). KEMRI-AUCC complies with the national guidelines for care and use of laboratory animals in Kenya developed by the Kenya Veterinary Association and the Kenya lab animal technicians association 1989. The KEMRI-AUCC which approved the study protocol has an assurance identification number A5879-01 from the Office of Laboratory Animal Welfare (OLAW) under the Kenyan department of health and human services. For purposes of livestock use, funds from the project were used to purchase animals to monitor RVFV seroprevalence and used for all experimental activities described in this study. The animal owners consented to the use of their animals. These animals were owned and maintained for the study by the project. The project bought 492 animals comprising 5 sentinel herds; two in Marigat, three in Ijara district (one in Kotile and 2 in Sangailu). The animals were left with the owners as part of their flocks but they were not allowed to sell or slaughter them because the project was monitoring the animals. The animals were reverted back to the owner at the end of the project activity. Any new borns born out of the tagged animals belonged to the farmers. We worked in collaboration with the department of veterinary services and veterinary doctors mandated by the government to do livestock sampling and research. The above terms were stipulated well in an agreement between the farmers and the international Centre of Insect Physiology and Ecology (*icipe*), the hosting institution for the AVID Project Consortium. Human odor was collected from one of the authors, DPT, on worn stockinette and the mosquitoes are the subject of the experiment which responded to the stimuli on the stockinette. Entomological surveys were conducted away from homesteads and on community land as authorized by Community Elders after explaining the purpose of the study to them.

## Results

### Mosquito sampling with animal skin odors

**Figure 3:**
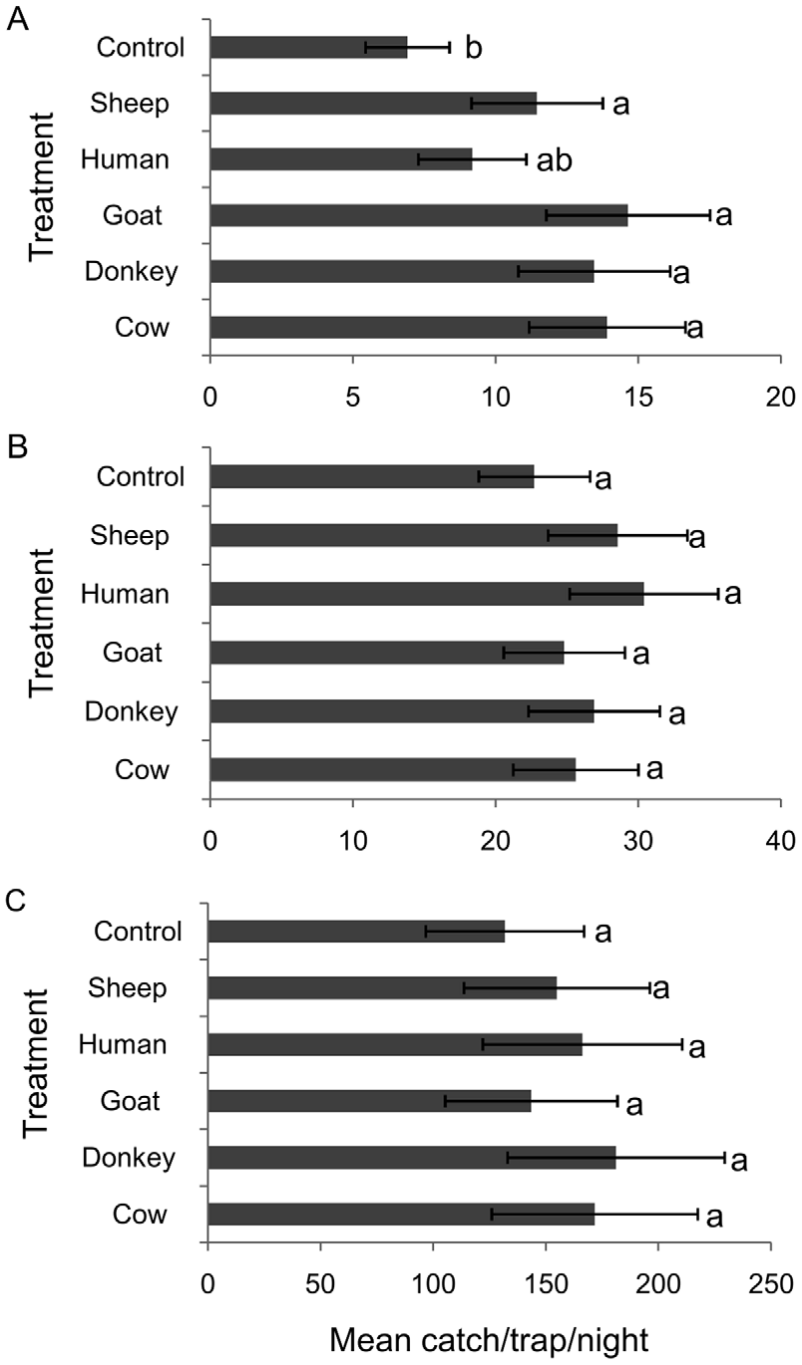
Mean daily captures of RVFV vectors in the different trap treatments in 11 replicate trials. (A) primary vectors (*Ae. mcintoshi* and *Ae. ochraceus*); secondary vectors comprising (B) total *Culex* spp.; (C) total *Mansonia* spp. Control, CO_2_-baited traps only; host treatments represent skin odors from each host type combined with CO_2_. Treatments followed by the same letters are not significantly different at P = 0.05 following generalized linear model (GLM) with negative binomial error structure and log link in R 2.11.0 software; Error bars indicate standard error of the mean.

Compared to the control CO_2_-baited trap, the addition of mammalian skin odors in all the treatment traps not only selectively targeted mosquitoes, but also significantly increased trap captures of primary RVFV vectors (*Ae. mcintoshi*/*Ae. ochraceus*) (p = 0.043); cow [IRR = 2.01, CI (1.14–3.57)], donkey [IRR = 1.95, CI (1.10–3.45)], goat [IRR = 2.12, CI (1.20–3.75)] and sheep [IRR = 1.66, CI (1.03–2.95)] (Figure 3). Notably, addition of human skin odors did not significantly increase mosquito captures over the control [IRR = 1.33 CI (0.74–2.38)]. We observed a similar pattern of mosquito captures for secondary vectors of RVFV, mainly *Culex* and *Mansonia* species in the mammalian skin-baited traps with a combination of CO_2_ and skin odors of these hosts although no significant differences were found compared to CO_2_ only (p = 0.872 for *Culex* spp. and p = 0.964 for *Mansonia* spp). Performance on captures of total *Culex* spp. (*Culex. pipiens*, *Cx. univittatus*, *Cx. poicilipes* and *Cx. bitaenorryhnchus*) were: cow [IRR = 1.13, CI (0.70–1.82)], donkey [IRR = 1.18 CI (0.74–1.91)], goat [IRR = 1.09, CI (0.68–1.76)], human [IRR = 1.34, CI (0.83–2.15)], sheep [IRR = 1.26, CI (0.78–2.02)] and for total *Mansonia* spp. (*Mansonia. uniformis* and *Mn. africana*): cow [IRR = 1.30, CI (0.62–2.75)], donkey [IRR = 1.37, CI (0.65–2.90)], goat [IRR = 1.09, CI (0.52–2.30)], human [IRR = 1.26, CI (0.60–2.66)], sheep [IRR = 1.18 CI (0.56–2.48)]. We also observed a significant difference in the proportion of engorged mosquito (i.e., blood fed+gravid) recorded in the different treatments for both primary vectors (χ^2^ = 28.838, df = 5, p<0.001) and secondary vectors (χ^2^ = 122.897, df = 5, p<0.001). We found a higher proportion of engorged mosquito in baited traps containing CO_2_ plus animal odor relative to the control CO_2_ trap alone (Table 1).

**Table 1:** Proportion of engorged RVFV mosquito (blood fed+gravid) of total captures recorded in control traps (CO_2_ only) and combined lures having CO_2_ and crude skin odors from the different hosts evaluated with corresponding catch indices.

Treatment	Primary vectors (n)	Catch index	P-values	Secondary vectors (n)	Catch index	P-values
Control (CO_2_ only)	0.21 (76)	1	-	0.06 (3209)	1	-
Cow	0.46 (153)	2.17	<0.001	0.12 (4057)	2.15	<0.001
Donkey	0.24 (148)	1.16	0.703	0.09 (4277)	1.62	<0.001
Goat	0.30 (161)	1.45	0.207	0.12 (3497)	2.15	<0.001
Sheep	0.34 (126)	1.62	0.069	0.10 (3829)	1.79	<0.001
Human	0.20 (101)	0.94	0.987	0.08 (4101)	1.44	0.001

Primary vectors (*Ae. mcintoshi* and *Ae. ochraceus*); secondary vectors (total counts of *Culex* and *Mansonia* spp.); n in parenthesis represents total mosquito captures recorded per treatment. P-values based on pair-wise comparison to CO_2_ following chi-square goodness-of-fit in R 2.11.0 software.

### Chemical identification of animal skin odors

**Figure 4:**
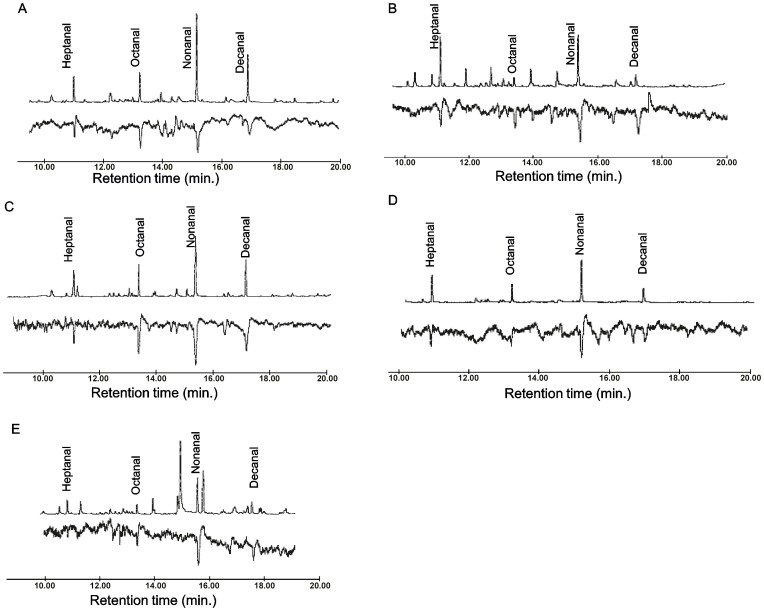
Representative GC-EAD profiles using wild caught adult female *Ae. mcintoshi* to the different host odors. (A) Cow (B) Donkey (C) Human (D) Goat (E) Sheep. Upper traces are FID (chemical profile) of the respective host odor and lower traces are EAD responses. Regardless of host type, similar responses to the four aldehydes (whose peaks are labeled in the uppermost trace) were reproducibly recorded not only in this species but in *Ae. ochraceus* and diverse species of *Culex* and *Mansonia* which are secondary RVFV vectors (n = 3). (Scale bar in all the GC-EAD runs, 1 mV.)

By comparing GC-EAD patterns, we observed four identical peaks in volatiles from each host that consistently elicited antennal responses from the different mosquito species (Figure 4). Using GC-MS, we identified the components representing these peaks as heptanal, octanal, nonanal and decanal and confirmed their identities by comparing retention times and fragmentation patterns with authentic standards. The total amount of these aldehydes in the volatiles varied with the host *viz*: cow, 29–43%; goat, 45–56%; donkey, 35–63%; sheep, 26–44%; and human, 18–40%.

### Development and evaluation of an improved RVFV mosquito vector trapping system

To maximize sensitivity of the trapping system to target selectively RVFV mosquito vectors, we first compared mosquito trap captures in preliminary field dose-response assays using CO_2_-baited CDC trap without a light bulb combined with individual synthetic EAG-active compounds to the control trap baited with CO_2_ alone. Since our chemical analysis showed that the total amount of aldehydes varied within and between replicates of individual host odors, we therefore formulated a blend (Blend F) from the four aldehydes based on the doses of individual components that elicited optimal attraction in the preliminary field assays (Figure S1). These were heptanal, 2 mg/ml; octanal, 0.5 mg/ml; nonanal, 0.1 mg/ml; and decanal, 0.1 mg/ml (Figure S1). We also constituted representative blends reflecting the mean ratio of occurrence of these aldehydes in each of the animals: Blend A (cow); Blend B (human); Blend C (goat); Blend D (sheep); Blend E (donkey) (Table S4). In subsequent dose-response field assays, we then compared the attractiveness of these blends (A–F) to these individual components at their respective optimal doses. Overall for individual components, heptanal recorded the highest captures (61% increase at 2 mg/ml), followed by nonanal (44% increase at 0.1 mg/ml, decanal (36% increase at 0.1 mg/ml and octanal (34% increase at 0.5 mg/ml) (Table 2). However, these increases were not significantly different from the control captures (Table 2).

**Table 2:** Captures of primary RVFV mosquito vectors (*Ae. mcintoshi* and *Ae. ochraceus*) recorded at different optimal synthetic component doses and a blend in 9 replicate field trials.

Aldehyde treatments	Number caught	IRR (95% CI)	P-values
Blend A	104	1.30 (0.70–2.43)	0.408
Blend B	85	1.06 (0.57–2.00)	0.850
Blend C	96	1.20 (0.64–2.24)	0.567
Blend D	88	1.10 (0.59–2.06)	0.766
Blend E	91	1.14 (0.61–2.13)	0.686
Blend F	258	3.23 (1.76–5.91)	<0.001
2 Heptanal	129	1.61 (0.87–2.99)	0.128
0.5 Octanal	107	1.34 (0.72–2.49)	0.358
0.1 Nonanal	115	1.44 (0.77–2.67)	0.250
0.1 Decanal	109	1.36 (0.73–2.54)	0.328

Incidence rate ratio (IRR) and corresponding 95% confidence interval (CI) and P-values; P-values based on comparison to CO_2_ following generalized linear model (GLM) with negative binomial error structure and log link in R 2.11.0 software; number in front of each compound represents the optimal dose of each component evaluated in mg/ml.

We found that there was significant treatment effect on mosquito captures of primary RVFV vectors (*Ae. mcintoshi* and *Ae. ochraceus*), (χ^2^ = 104.81, df = 10, p = 0.003) and for total mosquito captures (χ^2^ = 107.28, df = 10, p = 0.01). Expectedly, we observed increased captures of total primary RVFV vectors in traps baited with CO_2_ combined with the optimal doses of each component and blends representing the mammalian odors (Blends A–E) although these captures were not significantly different from the control (Table 2). Interestingly, Blend F formulated from the optimal attractive doses of individual components performed far better than any of the representative mammalian blends (A–E) and the individual components (Table 2), trapping significantly three-fold more of the primary RVFV vectors than the control trap [(IRR = 3.23, CI (1.76–5.91)] (Table 2). Equally interesting, we found a significant increase in total mosquito captures including *Culex* RVFV secondary vectors relative to the control [IRR = 2.35, CI (1.33–4.18)]. An equally interesting finding we observed, was a significant difference in the proportion of engorged mosquito in the total mosquito captures for the optimal compounds and blends tested (χ^2^ = 56.174, df = 10, p<0.001) relative to the control (Table 3). Similarly as found for animal crude odors, we observed a clear pattern of a higher proportion of engorged mosquitoes in all traps containing CO_2_ plus single compounds/blends relative to the control (Table 3).

**Table 3:** Proportion of total engorged RVFV mosquito (blood fed+gravid) of total captures recorded in control traps (CO_2_ only) and combined lures having CO_2_ and optimal single compounds/blends evaluated and corresponding catch indices.

Treatment	Optimal dose (mg/ml)	Proportion (n)	Catch index	P-values
Control (CO_2_ only)		0.05 (210)	1	-
Heptanal	2	0.19 (325)	3.58	<0.001
Octanal	0.5	0.14 (291)	2.69	0.002
Nonanal	0.1	0.08 (320)	1.43	0.271
Decanal	0.1	0.09 (299)	1.66	0.152
Blend A		0.08 (237)	1.45	0.326
Blend B		0.07 (268)	1.28	0.523
Blend C		0.13 (243)	2.51	0.007
Blend D		0.07 (237)	1.29	0.518
Blend E		0.15 (212)	2.88	0.001
Blend F		0.14 (494)	2.63	0.001

n in parenthesis represents total mosquito captures recorded per treatment. P-values based on pair-wise comparison to CO_2_ following chi-square goodness-of-fit in R 2.11.0 software.

## Discussion

RVF represents a looming health threat to various parts of the world [2]. The virus continues to circulate among animals and humans in many areas, both during intermittent epidemics/epizootics and IEPs [4], [23]. Thus, the presence and intimate association of capable vectors and susceptible hosts such as livestock, humans in the same ecosystem as in Marigat district and Ijara district of North-Eastern Kenya, may help sustain this virus. As such, low numbers of vectors may be required for virus maintenance in a population of large susceptible vertebrate hosts in the environment as in the IEP. Spread and introduction of infection to new areas over long distances via movement of infected vectors remains plausible.

In this study, we have demonstrated overwhelmingly through robust field-guided and chemical analyses that mammalian skin odors attract RVFV vectors. Our findings concur with published literature highlighting the importance of animal skin odors in mosquito attraction [17], [24]–[26]. Among the animal hosts examined, primary RVFV vectors (comprising *Ae. mcintoshi* and *Ae. ochraceus*) showed a bias towards skin odors of animal hosts compared to humans in agreement with previous observations showing that during the 2007/2008 RVF outbreak in North Eastern Kenya these two mosquito species accounted for approximately 80% of positive pools of mosquitoes sampled in the field [8]. Furthermore, this observation supports the epidemiology of RVF as a zoonosis with circulation mainly among vertebrate animals which serve as efficient amplifiers of the virus and only incidental transmission to humans [10], [27]. For the secondary *Culex* and *Mansonia* vector species, although there was a marked effect of odors on mosquito captures, there was no clear pattern of preference in their attraction among the animal host odors examined. This may suggest a more widespread feeding pattern of these vectors and cement their role as bridge vectors in the extension of the disease to humans.

Our results show that the amounts of host skin-derived aldehydes varied within and between hosts, and further that given the commonality of the host skin-derived volatiles; these profiles may extend to related mammals attractive to RVFV mosquito vectors including wildlife. Host skin-derived aldehydes seem to play an important role in the attractiveness of RVFV mosquitoes, but better as a blend rather than as individual components. This pattern has been observed for other mosquito species and tsetse flies in combination with other chemical compounds [28]–[30]. Our data show that blends of synthetic aldehydes representing different host animals worked in combination with CO_2_ to attract RVFV mosquito vectors differentially. Because these captures were comparable to those we obtained with the crude skin volatiles, our data lends strong support for the role of aldehydes in mosquito attraction to host animals. However, a striking feature of our evaluation of aldehyde blends is the higher attractiveness for the altered blend formulated from doses of the individual aldehydes that elicited optimal attraction of vectors, clearly demonstrating its potential for practical use in monitoring RVFV mosquito vector populations. From this observation we believe that the ratios and release rates of aldehydes from an individual animal, irrespective of the host, determine its relative attractiveness in a herd. As such, some individuals in a herd would be relatively more attractive and serve as a sponge for RVFV mosquito vectors than others as demonstrated in this study when addition of individual compounds, at certain doses, reduced mosquito trap capture while other blends of components significantly increased trap capture. Nonetheless, because of the commonality of the host chemical signature required for attraction, the host-vector interaction would appear to favor the mosquito vector allowing it to find and opportunistically feed on a wide range of mammalian RVFV hosts. Hence, the natural survival of this arboviral pathogen is tightly bound to the success of this olfactory-based activity which these mosquito vectors use in seeking and feeding on multiple hosts. It is possible that microbial endogenous breakdown products of surface lipids may be the likely origin of these compounds on animal skin or from exogenous deposition via contact with foreign substances [31], which would require additional research. Furthermore, the influence of skin bacteria on mosquito attraction has recently been highlighted [32].

A number of studies have reported the importance of aldehydes in the sensory ecology of mosquitoes [26], [33], [34] and various blood feeding arthropods, including ticks [35], triatomine bugs [36], and tsetse flies [37], [38]. For mosquitoes in particular, their roles in the balance of attraction and inhibition have been suggested [39], [40] mainly in laboratory assays and with limited efforts in field settings. Nonetheless, in a recent study CO_2_ was reported to synergize nonanal to increase trap captures of *Culex* mosquito vectors of West Nile Virus [26]. Clearly, our data stresses a fascinating dose-dependent behavioral blend effect of four aldehydes as kairomones which in combination with CO_2_ significantly increases trap captures for RVFV mosquito vectors. Furthermore, our data also suggests that individually, heptanal, octanal and decanal can also be exploited in a similar manner to increase field captures of RVFV mosquito vectors.

Surveillance to monitor virus movement among vectors and hosts is crucial in informing public health decision makers for early warning and rapid response. Although trapping of adult female mosquito vectors remains a cornerstone of this strategy, efficient trapping tools for most of these RVFV vector species remain wanting especially during the IEP due to low sensitivity and non-specificity of currently available CO_2_-baited light trap. Moreover, enzootic transmission of arboviral diseases continues to occur at a low intensity among mosquito vectors in Kenya [23] and remain undetected. The development of the attractant blend described here circumvents the challenges by increasing captures not only for key RVFV vectors but also diverse mosquito species. This constitutes an important landmark as a practical effective population monitoring tool especially during the IEP so as to maximize trap captures for viral isolation in order to reveal the true burden of arbovirus circulating in affected communities. Furthermore, once mosquito vectors have been trapped and identified, their populations can be tracked to reveal important epidemiological parameters such as, population age structure, infection status, blood feeding patterns all culminating in assessing disease transmission risk. Improved arboviral vector surveillance equally requires knowledge of the mosquito population being sampled. Blood fed and gravid mosquito cohort because of their previous host encounter can be advantageous during surveillance as testing this cohort increases the likelihood of viral detections. Our results clearly indicate that traps baited with CO_2_ and animal skin odors both crude and synthetic compounds captured a higher proportion of engorged mosquitoes (blood-fed+gravid) than control traps. Our findings therefore, suggest the inclusion of attractive skin odors to CO_2_-baited traps for improved entomological surveillance.

The blend we have developed requires combination with CO_2_ supplied in the form of dry ice. This commercial source of CO_2_ is not only expensive but may readily be unavailable in remote areas, which may hamper mosquito collection. However, alternative forms of generating CO_2_ as an attractant using yeast have been evaluated [41], [42]. However, the efficacy of CO_2_ supplied as dry ice has been shown to increase mosquito captures significantly over that generated using yeast [43]. As the search for a suitable substitute for CO_2_ continues, a recent study identified 2-butanone as a mimic of CO_2_ activity following similar activation patterns in the odorant receptor neurons of mosquitoes [44]. The behavioral significance in terms of attraction in the natural habitat of mosquito vectors remains to be evaluated towards developing economical lures for use in trap-based mosquito surveillance especially in remote settings.

In summary, this work is the first comprehensive report of translational research utilizing chemical ecology to generate better tools for surveillance of RVFV vectors. We have employed field bioassay guided experiments in combination with conventional chemical ecology approaches to identify four compounds, heptanal, octanal, nonanal and decanal, which when tested in the field, singly and in blends, increased capture of a number of RVFV mosquito vectors. The most effective blend (Blend F), significantly improves attraction when used in conjunction with CO_2_ over that of the CO_2_-baited CDC traps alone, the latter currently used for surveillance and provides a clear improvement in our ability to monitor mosquito vectors especially during the IEP.

## Supporting Information

Figure S1 **Mean captures of primary RVFV vectors to different doses of compounds in preliminary field trials to establish optimal doses.** Numbers represent doses of each compound in mg/ml tested in combination with CO_2_; control, CDC trap without a light bulb baited with CO_2_ only; number of replicates, n = 3.
(TIF)

Table S1 **Approximate mean ratio of aldehyde components in the host odor profiles in GC-MS runs.**
(DOC)

Table S2 **Estimated mean amounts of aldehyde components released from the skin host volatiles.**
(DOC)

Table S3 **Preliminary trials conducted at** ***icipe*** **Duduville campus, Nairobi.**
(DOC)

Table S4 **Blend composition.**
(DOC)

Table S5 **Optimal dose of single compounds and estimated release rates together with blends evaluated.**
(DOC)
