# Supplementary figures and images for: Common Host-Derived Chemicals Increase Catches of Disease-Transmitting Mosquitoes and Can Improve Early Warning Systems for Rift Valley Fever Virus
Source: PLoS Negl Trop Dis. 2013 Jan 10;7(1):e2007. doi: 10.1371/journal.pntd.0002007 (PMC3542179; doi:10.1371/journal.pntd.0002007)

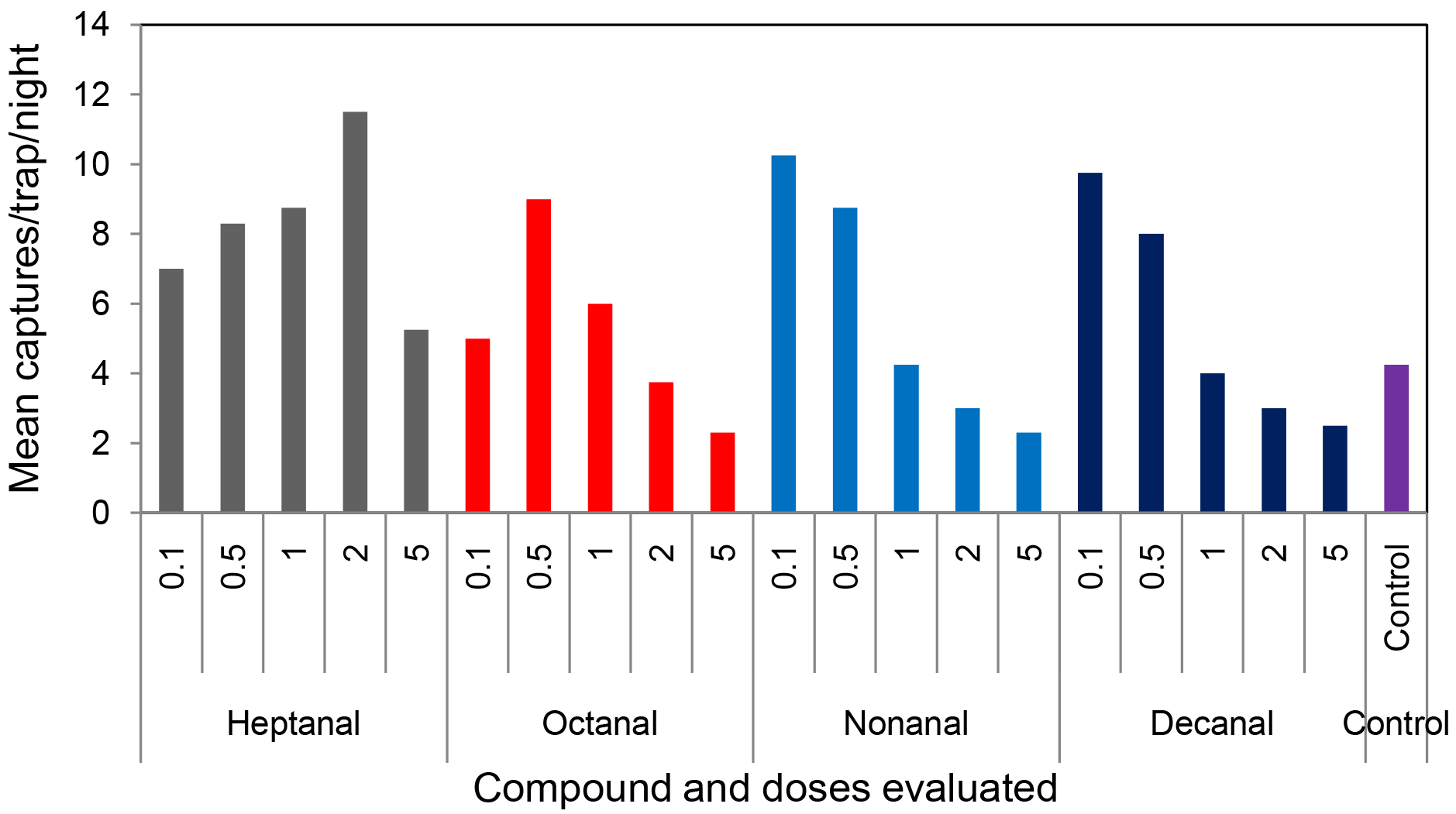

Supplement: Figure S1 — Mean captures of primary RVFV vectors to different doses of compounds in preliminary field trials to establish optimal doses. Numbers represent doses of each compound in mg/ml tested in combination with CO2; control, CDC trap without a light bulb baited with CO2 only; number of replicates, n = 3. (TIF) [file pntd.0002007.s001.tif]
