# Supplementary material for: Common Host-Derived Chemicals Increase Catches of Disease-Transmitting Mosquitoes and Can Improve Early Warning Systems for Rift Valley Fever Virus
Source: PLoS Negl Trop Dis. 2013 Jan 10;7(1):e2007. doi: 10.1371/journal.pntd.0002007 (PMC3542179; doi:10.1371/journal.pntd.0002007)
Supplement: Table S1 — Approximate mean ratio of aldehyde components in the host odor profiles in GC-MS runs. (DOC) [file pntd.0002007.s002.doc]

|  | **Compound** | | | |
| --- | --- | --- | --- | --- |
| **Host** | Heptanal | Octanal | Nonanal | Decanal |
| Human | 1 | 1 | 4 | 2 |
| Goat | 1 | 1 | 3 | 2 |
| Cow | 1 | 1 | 3 | 2 |
| Donkey | 4 | 1 | 4 | 2.7 |
| Sheep | 1 | 1 | 2 | 1.5 |
