# Supplementary material for: Common Host-Derived Chemicals Increase Catches of Disease-Transmitting Mosquitoes and Can Improve Early Warning Systems for Rift Valley Fever Virus
Source: PLoS Negl Trop Dis. 2013 Jan 10;7(1):e2007. doi: 10.1371/journal.pntd.0002007 (PMC3542179; doi:10.1371/journal.pntd.0002007)
Supplement: Table S2 — Estimated mean amounts of aldehyde components released from the skin host volatiles. (DOC) [file pntd.0002007.s003.doc]

|  | **Amount of compound released (µg/hr)** | | | |
| --- | --- | --- | --- | --- |
| **Mammalian host** | Heptanal | Octanal | Nonanal | Decanal |
| Donkey | 0.46 | 0.11 | 0.47 | 0.32 |
| Cow | 0.18 | 0.19 | 0.46 | 0.36 |
| Goat | 0.17 | 0.15 | 0.45 | 0.30 |
| Sheep | 0.19 | 0.12 | 0.34 | 0.28 |
| Human | 0.13 | 0.20 | 0.65 | 0.43 |
