# Supplementary material for: Common Host-Derived Chemicals Increase Catches of Disease-Transmitting Mosquitoes and Can Improve Early Warning Systems for Rift Valley Fever Virus
Source: PLoS Negl Trop Dis. 2013 Jan 10;7(1):e2007. doi: 10.1371/journal.pntd.0002007 (PMC3542179; doi:10.1371/journal.pntd.0002007)
Supplement: Table S3 — Preliminary trials conducted at icipe Duduville campus, Nairobi. (DOC) [file pntd.0002007.s004.doc]

| **Compound** | **Dose (mg/ml)** | **mosquito captures^1^ (n=6)** |
| --- | --- | --- |
| Heptanal | 1 | 10 |
|  | 5 | 8 |
|  | 10 | 0 |
| Octanal | 1 | 7 |
|  | 5 | 5 |
|  | 10 | 0 |
| Nonanal | 1 | 9 |
|  | 5 | 0 |
|  | 10 | 0 |
|  | 20 | 0 |
| Decanal | 1 | 7 |
|  | 5 | 0 |
|  | 10 | 0 |
| Control |  | 0 |

^1^Trap captures mainly *Culex quinquefasciatus* compared in CDC traps without a light bulb baited with individual doses of the compounds.
