# Supplementary material for: Common Host-Derived Chemicals Increase Catches of Disease-Transmitting Mosquitoes and Can Improve Early Warning Systems for Rift Valley Fever Virus
Source: PLoS Negl Trop Dis. 2013 Jan 10;7(1):e2007. doi: 10.1371/journal.pntd.0002007 (PMC3542179; doi:10.1371/journal.pntd.0002007)
Supplement: Table S4 — Blend composition. (DOC) [file pntd.0002007.s005.doc]

|  | **Compound (mg/ml)** | | | |
| --- | --- | --- | --- | --- |
| **Blend** | Heptanal | Octanal | Nonanal | Decanal |
| Blend A | 0.125 | 0.165 | 0.5 | 0.3 |
| Blend B | 0.25 | 0.33 | 1 | 0.6 |
| Blend C | 0.2 | 0.2 | 0.5 | 0.375 |
| Blend D | 0.5 | 0.125 | 0.5 | 0.5 |
| Blend E | 0.75 | 0.25 | 1 | 0.375 |
| Blend F | 2 | 0.5 | 0.1 | 0.1 |
