# Supplementary material for: Common Host-Derived Chemicals Increase Catches of Disease-Transmitting Mosquitoes and Can Improve Early Warning Systems for Rift Valley Fever Virus
Source: PLoS Negl Trop Dis. 2013 Jan 10;7(1):e2007. doi: 10.1371/journal.pntd.0002007 (PMC3542179; doi:10.1371/journal.pntd.0002007)
Supplement: Table S5 — Optimal dose of single compounds and estimated release rates together with blends evaluated. (DOC) [file pntd.0002007.s006.doc]

| **Compound** | **Optimal dose (mg/ml)** | **Release rate (µg/hr)** |
| --- | --- | --- |
| Heptanal | 2 | 0.14 |
| Octanal | 0.5 | 0.12 |
| Nonanal | 0.1 | 0.13 |
| Decanal | 0.1 | 0.15 |
| Blend A |  | 0.16 |
| Blend B |  | 0.05 |
| Blend C |  | 0.12 |
| Blend D |  | 0.13 |
| Blend E |  | 0.15 |
| Blend F |  | 0.14 |
